# Dehumanizing air travel: a scoping review on accessibility and inclusion of people with disabilities in international airports

**DOI:** 10.3389/fresc.2024.1305191

**Published:** 2024-08-27

**Authors:** David Gotti, Ernesto Morales, François Routhier, Jonathan Riendeau, Ahmed Hadj Hassen

**Affiliations:** ^1^ School of Rehabilitation Sciences, Laval University, Québec City, QC, Canada; ^2^ Centre for Interdisciplinary Research in Rehabilitation and Social Integration, Centre Intégré Universitaire de Santé et de Services Sociaux de la Capitale-Nationale du Québec, Québec City, QC, Canada

**Keywords:** accessibility, airport, inclusive, people with disabilities, participation

## Abstract

**Introduction:**

Worldwide, one in six individuals live with a disability. Many people continue to experience disabling situations, particularly when travelling. Travel can be an important part of the lives of many people, including people with disabilities. Barriers to accessing air travel can lead to a reluctance to travel for these potential passengers. As early as the flight planning stage, options to facilitate accessibility are limited. At airports, passengers must travel long distances in areas where navigation is complex, and accessibility limited. Furthermore, few aircraft are accessible. The travel experience can have adverse effects on the health of people with disabilities. Practices are sometimes not inclusive, not always available, and may not address the full spectrum of possible needs. The objective of this study is to provide a state of art on accessibility features available to people with disabilities in the busiest international airports.

**Methods:**

A scoping review of practices in airport accessibility was done. A search strategy was deployed in 4 databases (Avery index to architectural periodicals, Medline, CINAHL, and Web of science). The official websites of the 35 busiest airports were exhaustively consulted. All information regarding accessibility measures and inclusive services were extracted.

**Results:**

31 scientific articles, out of 3,640 screened, and all extracted data from airports’ website were considered for synthesis. While efforts are made in major airports, there is a great inconsistency in accessibility features. Both facilitators and challenges are encountered by people with disabilities at every stage of air travel. These stages include journey planning; getting to and from the airport, obtain human assistance, navigate in the premises, check in, security screening, using facilities, boarding and disembarking, customs and immigration procedures, and luggage management.

**Discussion:**

Services need to be extensively planned, placing a significant burden on passengers. The disability-centric perspective disregard passengers’ unique needs and capabilities, leading to a sense of dehumanization. The complexity of airport organizations, shared responsibilities, limited communication, training challenges can deter accessibility initiatives and create discomfort during travel. This study is the first step of a broader project supported by Canadian Accessibility Standards, focusing on enhancing inclusive accessibility in Canadian airports.

## 1 Introduction

In 2021, approximately one in six person had one or more disabilities leading to disabling situations (1). In Canada, this phenomenon is particularly salient, with more than one in four people living with a disability (2). To address this situation, the Government of Canada proposed several programs and laws focused on equal rights and opportunities for people with disabilities (PwD) (3). Despite this desire for equity, many people still experience disabling situations when carrying out their daily lives, particularly when it comes to travel. Air travel can hold a significant meaning for the social, productive and leisure of everyone, including people living with a disability or disabilities (4, 5).

The many barriers encountered during air travel bring a sense of discrimination and reluctance to travel for PwD (4, 6, 7). From the planning stage onwards, there is no guarantee that the flight provider will be able to accommodate the individual's unique needs. Most airlines offer accessibility information, but the level of detail of the information is insufficient to make an informed decision about travel (8). Often, travellers are forced to contact service providers by phone and give highly personal health information (5, 6). Moreover, few aircraft are accessible. Aircraft configurations and restricted spaces force staff to resort to means perceived as dehumanizing. For example, people in wheelchairs often have to change seats, be manipulated over armrests, board long before other passengers or disembark last. In addition to discomfort and pain, these measures can exacerbate a sense of segregation for these passengers (4, 5). In some cases, boarding staff's perception of a person's degree of autonomy can result in denied boarding, if unaccompanied (5). Other barriers are related to airport facilities. In particular, passengers often have to travel long distances in complex environments. These features can limit PwD’ ability to orient themselves and navigate the facilities (5, 7). At many stages, passengers must wait for long periods, in uncomfortable areas, and/or adapted services are scarce (screening, passport, security, etc.). Often, few entertainment options, restaurants or waiting areas are accessible for PwD (6). Airport staff may also lack the knowledge to effectively support specific needs around and within the aircraft (4, 7).

Some airports are trying to mitigate these barriers. In Canada, for example, some airports offer individualized mobility assistance to passengers upon request (9–11). In Toronto, a GPS navigation system with verbal guidance is being tested to improve independence for people with visual impairments (12). Also, a mobile application is available for people with neurodiversity, to guide them step-by-step through the various common procedures at the airport (13). Other services are offered elsewhere. For example, at Hartsfield-Jackson airport in Atlanta, USA, or Haneda International in Tokyo, Japan, quiet areas are available for passengers with developmental, intellectual disabilities or mental health issues (14, 15).

However, while efforts are being made to improve the experience of passengers with disabilities, they can raise other issues. Travel planning is much more important for PwD than for other passengers. Many airport services need to be booked 72–48 h in advance, and there is no guarantee that they will still be available, even after a ticket has been purchased. Passengers must constantly check and anticipate the suitability of the services offered for their specific needs (4, 6, 8). Challenges experienced by passengers with disabilities can lead to adverse effects on physical, psychological and emotional health (4, 5). Indeed, services are often designed according to a specific type of disability (e.g, motor disability, visual impairment), hardly representative of the complexity and diversity of factors that influence the needs that PwD may have (16). This issue is exacerbated by the predominant vision of adaptation rather than inclusiveness in airport accessibility. Indeed, services are primarily aimed at overcoming environmental barriers. These adaptations are not always comfortable or respectful of individual particularities. The systematic use of assistance staff, “segregated” circulation channels and means different from other passengers can create a feeling of marginalization, even humiliation, for passengers with disabilities (5, 7).

Both PwD and airport service stakeholders insist on the importance of developing and updating the measures deployed to improve the experience of passengers living with disability situations. However, it is not very clear what exists today in terms of accessibility practices at international airports around the world. Therefore, the aim of this study is to explore and map existing accessibility and inclusive practices for PwD at international airports, in order to identify gaps and shortcomings.

## 2 Methods

To address the main objective, a scoping review was carried out through various stages using several methodological guides (17–21).

### 2.1 Research strategy

The research was developed in order to identify all the scientific literature and grey literature relevant to the research topic.

#### 2.1.1 Scientific literature

A professional librarian, affiliated with Laval University, helped identify the scientific literature relevant to the research question. Her expertise in review methods and specialization in the healthcare field ensured the accuracy, comprehensiveness and relevance of the search. An initial list of keywords and descriptors was identified via eight articles from the preliminary review. The terms were refined and validated by several members of the research team (DG, PhD student; EM and FR, professors). The terminology used for the research is presented in Appendix A. The search strategy was deployed in four databases (Avery's index of architectural periodicals, MEDLINE, CINAHL and Web Of Science). The databases were chosen to reflect the diversity of fields relevant to accessibility and participation: health (CINAHL, Medline), planning and architecture (Avery index of architectural periodicals) and multidisciplinary (Web Of Science). Endnote software (22) was used to collate all references from the databases. The databases were consulted on November 2, 2022.

#### 2.1.2 Selection of scientific articles

Given the exploratory nature of the scoping review, the criteria did not consider study designs. The selection process was facilitated by Covidence software (23). Covidence is a web-based collaboration software platform that streamlines the production of systematic and other literature reviews. Two members of the research team (DG; JR, research professional) made the initial selection based on titles and abstract. Of the articles selected, they (DG; JR) consulted the full texts to validate the presence of the inclusion and exclusion criteria, presented in Table 1.

**Table 1:** Scoping review inclusion and exclusion criteria.

Inclusion criteria	Exclusion criteria
Focuses on people with disabilities or living with handicaps. Relates to accessibility and inclusion. Pertains to measures or studies at airports Aims at effects on participation, accessibility, use, inclusion, desire to travel or suitability of the physical environment.	Is a market analysis Is a statistical analysis of air traffic Pertains only to freight, cargo, military or aerospace personnel Focuses exclusively on the issues and effects of profitability, traffic, equipment, environment or flight management. Other type of publication: advertising, protocol, magazines, books, opinion pieces. Written in a language other than French or English.

#### 2.1.3 Grey literature

At the same time, the websites of the 35 busiest international airports, as well as all Canadian international airports, were consulted. To obtain a list of these airports (Table 2), pre-pandemic air traffic and traffic statistics were used (24, 25). The data available for the pandemic period varies greatly from previous years, given the unequal restrictions on activities between different airports (26). Previous data from the COVID-19 pandemic are therefore more representative of the usual activities of international airports. Each website was consulted in two stages. Firstly, a free manual search was carried out to extract information from sections dealing with services, policies and information on accessibility or inclusion. Then, several keywords, such as “accessibility”, “special” or “barrier-free” (see Appendix B) were entered into each website's search engine to obtain data not captured by the first stage. External links to guides or third-party organizations were also considered.

**Table 2:** World's and Canada's busiest international airports (Port Authority of New York and New Jersey, 2019; statistics Canada, 2021).

Rank (2019)	Country, city	Airport
International		
1	United States, Atlanta	Hartsfield-Jackson Atlanta International Airport
2	China, Beijing	Beijing Capital International Airport
3	United States, Los Angeles	Los Angeles International Airport
4	United Arab Emirates, Dubai	Dubai International Airport
5	Japan, Tokyo	Tokyo International (Haneda) Airport
6	United States, Chicago	O'Hare International Airport
7	United Kingdom, London	Heathrow Airport
8	China, Shanghai	Pudong International Airport
9	France, Paris	Aéroport de Paris-Charles de Gaulle / Orly
10	United States, Dallas	Dallas/Ft Worth International Airport
11	China, Guangzhou	Guangzhou Bai Yun International Airport
12	Netherlands, Amsterdam	Amsterdam Airport Schiphol
13	Hong Kong SAR	Hong Kong International Airport
14	Korea, Republic of, Incheon	Incheon International Airport
15	Germany, Frankfurt	Flughafen Frankfurt/Main
16	United States, Danver	Denver International Airport
17	India, New Delhi	Indira Gandhi International Airport
18	Singapore	Singapore Changi Airport
19	Thailand, Bangkok	Suvarnabhumi International Airport
20	United States, New York	John F. Kennedy International Airport
21	Malaysia, Kuala Lumpur	KL International Airport
22	Spain, Madrid	Aeropuerto de Adolfo Suarez Madrid-Barajas
23	United States, San Francisco	San Francisco International Airport
24	China, Chengdu	Chengdu Shuangliu International Airport
25	Indonesia, Jakarta	Soekarno-Hatta International Airport
26	China, Shenzhen	Shenzhen Baoan International Airport
27	Spain, Barcelona	Aeropuerto de Barcelona-El Prat
28	Turkey, Istanbul	Istanbul International Airport
29	United States, Seattle	Seattle-Tacoma International Airport
30	United States, Las Vegas	Harry Reid International Airport
33	Mexico, Mexico City	Aeropuerto Internacional de la Ciudad de Mexico “Lic Benito Juarez"
34	United States, Charlotte	Charlotte Douglas International Airport
35	Russian Federation, Moscow	Sheremetyevo International Airport
Canada		
1	Canada, Toronto	Toronto/Lester B Pearson International, Ontario
2	Canada, Vancouver	Vancouver International, British Columbia
3	Canada, Montréal	Montréal/Pierre Elliott Trudeau International, Quebec
4	Canada, Calgary	Calgary International, Alberta
5	Canada, Edmonton	Edmonton International, Alberta
6	Canada, Ottawa	Ottawa/Macdonald-Cartier International, Ontario
7	Canada, Winnipeg	Winnipeg/James Armstrong Richardson International, Manitoba
8	Canada, Halifax	Halifax/Robert L Stanfield International, Nova Scotia
9	Canada, Victoria	Victoria International, British Columbia
10	Canada, Québec	Québec/Jean Lesage International, Québec

### 2.2 Data extraction

Extraction was performed according to the methodological specifics of scoping reviews (18). Descriptive data about study design, location, population and context were extracted. The analysis of this data has particular relevance to the object of study, given the diversity of socio-economic, political and cultural influences at different international airports. It helped provide information on the nature and distribution of the studies included (17). Additionally, Bronfenbrenner (27) ecological model's categories, relevant to the object of study (microsystem, mesosystem and macrosystem), were included in the exaction grid (see Appendix C), to ensure that the different levels of contextual factors impacting airports’ accessibility were taken into account. Finally, the grid included a section dedicated to the researcher's reflections and notes that may influence the analysis. Two members of the research team (DG, JR) carried out 10% of the extraction, until consensus was reached, to ensure the comprehensiveness and relevance of the extracted data (18).

### 2.3 Data analysis

Data were subject to thematic content analysis (17, 18, 28–30). This type of analysis is recommended for exploratory studies where data is limited (28, 31). The analysis was carried out using a mixed deductive and inductive approach. Bronfenbrenner (27) model's categories were used to group identified themes and facilitate their analysis (28). Grouping the codes by level of influence has made it easier to identify the main themes, ensuring that they are complementary and mutually exclusive. Identified themes are presented in Table 3. In a subsequent step, the data was linked to the relevant travel stages, to better visualize the traveler's experience over time. Three members of the research team (DG, EM, FR) were involved in data analysis, using Nvivo software (32). Several reduction steps were performed to reduce the number of codes identified and refine the data synthesis.

**Table 3:** Categories, themes and codes used in the thematic content analysis.

Categories	Themes	Codes
Microsystem	Airport physical environment	Interior installations
		Exterior installations
		Vehicles (inside)
		Key step areas
	Airport social environment	Personal assistant
		Human assistance (airport)
		Other passengers
		Children and families
	Airport services infrastructures	Personal care facilities
		Stores
		Specialized rooms and spaces
		Restaurants
		Emergency resources
	Personal equipment	Luggage
		Medical devices and supplies
		Baby and children equipment
		Assistive technology, communication and information
	Personal factors	Health
		Disability
		Knowledge
Mesosystem	Organizational culture	Accessibility committee
		Values and beliefs
		Perceived utility
	Staff management	Communication
		Skills and expertise
		Training
	Internal operations	Airport regulations
		Objectives and quality standards
		Advice given to passengers
	Services management	Human assistance management
		Key travel steps services
		Customer service
		Transportation programs
		Other services and programs
Macrosystem	External partners	External partners
	Practice guidelines	Practice guidelines
	Laws and regulations	Laws and regulations
	Social disability perspective	Social disability perspective
Needs	Meaning	Leisure and pleasure
		Access to health services
		Maintaining social relations
	Autonomy	Pursuing interests and values
		Advocate
		Equity
		Independence
	Competency	Communicate
		Efficiency and time
		Flexibility
		Planning and anticipation
	Safety	Health (physical, mental and emotional)
		Emergency
		Dignity

## 3 Results

### 3.1 Scientific articles included

**Figure 1:**
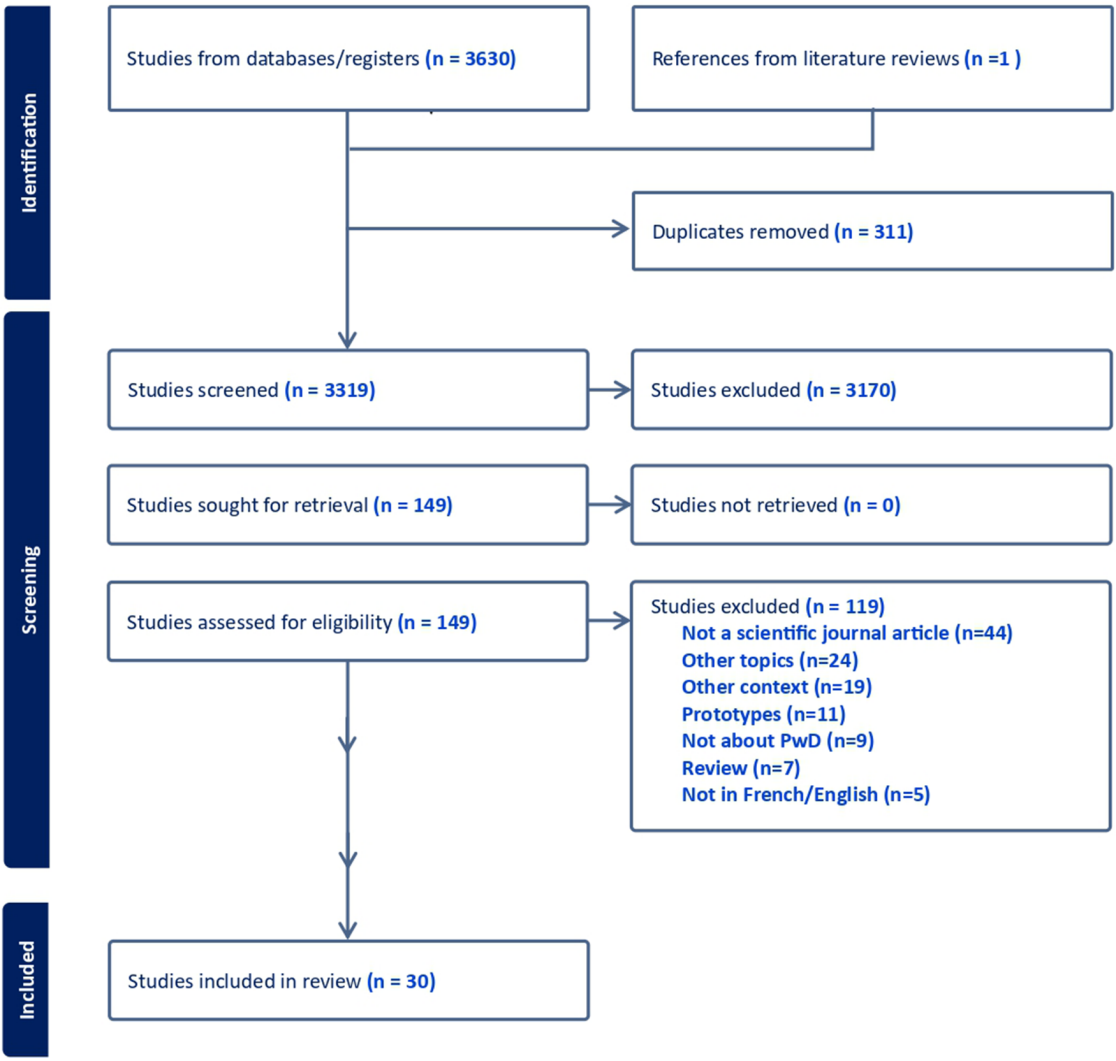
Scientific literature selection process: PRISMA diagram. Licensed under CC BY 4.0, Page et al. (33).

The article selection process is shown in a PRISMA diagram (Figure 1) (33). The database search strategy identified 3,319 unique results. After the selection process, 30 scientific articles were retained for analysis. Systematic reviews were not retained, in order to prioritize first-source literature. The quality of scientific articles was not assessed, and they were not discriminated on the basis of design. The majority of studies were based on qualitative research designs (*n* = 20), while others used quantitative (*n* = 7) or mixed methods (*n* = 4). Table 4 present included studies characteristics and distribution.

**Table 4:** Included studies characteristics.

Study characteristics		Number of studies
Origin of the study	Europe	15
	North America	11
	Asia	5
Year of publication	2009	2
	2010	4
	2011	1
	2012	2
	2014	2
	2015	1
	2016	1
	2017	1
	2018	2
	2019	3
	2020	5
	2121	4
	2022	3
Aim of the study	Highlight accessibility issues	16
	Evaluate accessibility and propose solutions	11
	Address accessibility issues	4
Studied population	PwD	22
	Older adults	4
	Employees	4
	Accompanying person	1

### 3.2 Included grey literature

Table 2 shows all the airports from which the information available on the websites was extracted. Airline web pages were also included when referenced by the selected airport websites.

The desire to support travel opportunities for PwD is not a new topic in airport industry environments. The literature reviewed shows that both in the field and in research, measures and recommendations are being deployed to support the experience of passengers living with disabilities. Results are presented to highlight relevant facilitators and barriers at each step of the journey. Also, additional aspects affecting travel experiences are presented.

### 3.3 Travel steps and activities

PwD travel primarily for leisure, for pleasure (34, 35). For many, travel is an enriching experience, contributing to self-fulfillment and quality of life (34). Like other means of transport, air travel can also be instrumental in maintaining social, family or friendship ties (34, 36). The desire to support travel opportunities for PwD is not a new topic in airport industry environments. To assess the impact of practices for the travel experience of passengers with disabilities, it is important to consider the complexity and scope of travel. Each stage of the journey represents a distinct experience, reality and challenge. To have a more comprehensive understanding of the results of this scoping review, they will be presented according to the different stages and activities that are required for travel.

#### 3.3.1 Accessing the information needed to plan the journey

For PwD, planning is required to make the journey possible, especially to anticipate longer steps and potential unforeseen circumstances that could make the trip impossible (37). This is all the more important for PwD, who may need more time and accommodation (37, 38). Airplane passenger have expressed the need to feel safe in an airport and during flight, both during the normal process and during emergencies (34, 39, 40). Health is also a central concern. It is therefore essential for PwD to have easy access to guidelines for planning, anticipating and managing their health while travelling (38).

Airport and airlines websites are the primary information channels for passengers. The vast majority of airport websites offer a description of their accessible services. Where relevant, airports refer to airlines websites or contact information for travel steps where they are responsible for accessibility.

Several airports, such as Dubai and Hong Kong airports, apply recognized web accessibility guidelines for their website (41, 42). Regulations and guides are available to guide website design. European airports adhere to the European Union Council's resolution on the accessibility of public websites and their content (43). They must also comply with the law on information society services and e-commerce. Notably, Aena (operator of Spanish airports), is aiming for a Double-A validation certificate for website accessibility, as recommended by the Web Accessibility Initiative (WAI) of the World Wide Web Consortium (W3C). Similarly, in the United States and Canada, public organizations are obliged to make the content of their web pages accessible (44, 45). They must ensure equitable access to information, in accordance with the principles of Web Content Accessibility Guideline 2.0 (46). This guide presents practical ways of creating accessible web content for PwD and aims to ensure that online information respects key usability principles. More specifically, information must be perceivable, usable, understandable and compatible with assistive technology tools (38). In this sense, the sites allow the use of screen readers and offer a wide range of customization options: alternative text for image content, adjustable font sizes, modification of contrasts, accent modes on text zones, etc. At Heathrow airport, UK, AbilityNet, a group of web accessibility experts founded by the Foundation for Communication for the Disabled and the Computability Centre, was consulted on the development of the website (47).

However, website accessibility is heterogeneous. Guidelines and regulations are not always followed, and in some cases, PwD may have to ask another person to use the website or contact customer service directly. In that case, it is not uncommon for people to have to pay more for their trip (48). Additionally, finding accurate information about accessibility is usually difficult and time-consuming (49).

Some Airports, such as Toronto's Pearson Airport provide additional accessible information material, to help with planning, through the “MagnusCard” mobile application. Notably, this information enables people with autism to plan the trip with greater ease and reduce the stress of the unfamiliar (13). Cerdan Chiscano (50) also recommend using dedicated material to help people with autism prepare the trip.

#### 3.3.2 Getting to and from the airport

Accessibility to and from airports largely depends on the accessibility of the means of private or public transport used. At airports such as Calgary, Dallas and Singapore, some public buses are equipped to accommodate wheelchair users (36, 51, 52). In Singapore, a tactile guidance system, real-time interactive maps and audio announcements are used to guide PwD in the subway (51). In some cases, such as Dallas-Ft Worth or Heathrow, shuttles between terminals are fully accessible to wheelchair users and passengers with reduced mobility (52, 53). Other partner organizations sometime offer accessible transportation options, as in Denver's airport, where wheelchair-accessible vans and hand-operated cars are available for rental (54). On another note, to facilitate arrival at the airport, PwD may have access to dedicated parking spaces and fee exemption, such as in Incheon Airport, Republic of Korea (55).

However, few airports provide clear information on the proportion of services accessible, or their degree of availability. Cole et al. (49) highlight that there is a limited number of accessible vehicles and transportations. This can be problematic considering that in many cases, people are required to use several means of transport. For instance, passengers at Los Angeles Airport often need to do additional transit between remote terminals (56). Additionally, while most airports offer dedicated outdoor accessible points, high passenger traffic can reduce the efficiency of accessible transports (57). Finally, many airports insist that PwD arrive at the airport at least three hours in advance. While this recommendation applies to all passengers, in the case of PwD it may affect access to the services they require (53).

#### 3.3.3 Obtaining human assistance

Human assistance services are offered at all the international airports included in the analysis. Human assistant services are mainly designed to compensate for the lack of accessibility of the physical environment. They aim to support the mobility, communication and access to information for passengers with disabilities, through the different travel stages, as in Madrid Airport (58). Services are usually free, but some airlines may charge for assistance services or for enhanced services (59). In most cases, the assistants wear easily recognizable colour-coded jackets, so that they can be quickly identified by passengers. They are usually present in dedicated kiosks, close to the entrances and hotspots of airports. For example, at Indira Ghandi International Airport at Delhi, India, assistants trained to intervene with PwD are available at accessible check-in or information desks (60) and in Dallas, USA, staff are present directly in the terminals, 24 h a day, every day (52). Assistance can usually begin at any of the airport's access points. In Incheon, for example, pick-up can start as soon as you arrive at the subway station (55). When passengers are accompanied by an assistant, services often involve the use of a wheelchair, in addition to the attendant, regardless of disability (61).

PwD have to plan in advance for dedicated human assistance services, to minimize waiting times at the airport. In most airports, the airport, airlines and other contractors share the responsibility of providing assistance (62). Responsibilities vary between airports. In Calgary, for example, the airlines take charge of the passenger from check-in to boarding (36), whereas at O'Hare International Airport, the airlines coordinate services from the boarding gate (63). Early notice from passengers is often cited as a facilitator in the organization and speed of services offered. At Madrid international airport in Spain, for example, passengers requiring an attendant must contact the airline, to obtain a pass for the attendant (58). PwD can contact the airport using a request form, using a mobile application or directly on site at dedicated kiosks (36, 58). However, waiting times vary widely, depending on the number of passengers and the contact method. At Schiphol airport, Amsterdam, for example, a person arriving on site to request assistance, without having made a reservation, may have to wait up to 45 min. This waiting time does not exceed 30 min when the person has booked an assistant at least 48 h in advance (64).

To facilitate punctual assistance to people with invisible disabilities, 140 airports around the world are also partners in the “Hidden Disabilities Sunflower” program, such as Denver Airport. This program offers people with invisible disabilities the opportunity to wear a sunflower symbol, making it “visible” to those around them that they may need extra help. Some airports additionally train employees on certain invisible conditions or disabilities (61). At KL International, Malaysia, “The Butterfly Effect” service offers integrated assistance to people with invisible disabilities, particularly those with autism. People using the program receive additional documentation, are welcomed and guided around the airport's accessible infrastructure (65).

For airport, human assistant fleets requires intensive management. Competence and training are directly linked to service quality (66, 67). Wang and Cole (68) highlight the importance of basic health knowledge training for employees. Employees should be prepared to compose with the common situations PwD may encounter during travel. Additionally, the desire to help, patience, the ability to offer clear information, to relate and to support comfort are key determinants of service quality. At Schiphol in Amsterdam, Netherlands, assistants are given specialized training in safety, risk management and intervention (64). In Dubai, employees are trained according to Air Carrier Act's standards and a certification program is in place to ensure an adequate level of competence among employees that may interact with PwD (69).

Human assistance services present several considerable challenges for airports organizations. First, assistants have to contend with low wages, irregular schedules, and physically demanding conditions (62, 70). These conditions result in particularly high staff turnover. For instance, over 60% of assistants at Pittsburgh International have been with the company for less than 2 years. This situation affects service quality and adds stress to the remaining assistants (62).

Also, McCarthy (67) and Cole et al. (49) point to a significant gap in the training received by employees. Indeed, it is not uncommon for employees from different departments or management to receive different training, in terms of quality and comprehensiveness. In this study, participants employed in airports mentioned that they would like to see experts offer systematic, standardized content on services and inclusion for passengers with disabilities. They would also like training on certain types of disability, communication and transfers. Gaps in employee training can create a low sense of competence, which can negatively affect the relationship with the person being supported (67). Passengers’ experience corroborates this gap. For example, parents of children with disabilities are often reluctant to have them handled by assistants due to a lack of experience (35). This challenge is all the more pressing in airport contexts, where employees require extensive knowledge and skills, such as the ability to speak several languages (61).

In addition, coordination and communication between assistants is hindered by factors such as distance, complex environments, numerous requests for assistance and dependence on walkie-talkies, leading to gaps in real-time information, errors and a decline in service quality (62). Also, hierarchical tensions within the organization, negative perceptions of colleagues and negative interactions with passengers can reduce service quality (67).

Finally, the employee fleets of airports and airlines are under different management. Management processes should include efficient channels of communication between different parties, to improve the fluidity, efficiency and quality of services, particularly during service transitions (68).

#### 3.3.4 Getting around the airport

Most airports have physical installations designed to facilitate the mobility of PwD. The different type of mobility amenities in the physical environment is presented in Table 5.

**Table 5:** International airport's physical environment aspects used to improve mobility.

Physical environment features	Current practices in airports	Scientific literature recommendations
Signage	Large fonts Contrasting colours (e.g, Heathrow) Simple langage Anti-reflective coating (e.g, Paris)	Abundant use of clear and simple signs (40, 50). Contrasting colours Large fonts Static and dynamic signs Intelligent signs with multiple sensory modes Visual signals at boarding gates (71). Use of static, dynamic and intelligent signs (57)
	High luminosity day and night (e.g, Toronto)	Lighting should be provided for all times of the day and night (71)
Outdoor		
Meeting points	Placed near the entrance (e.g, Madrid)	
Parking	Accessible, reserved, and close to entrance parking spaces (e.g, Atlanta)	Van-accessible spaces (49)
Curb	Accessible drop-off areas (e.g, Québec)	
Indoor		
Entrance door	Large entrance, Sliding doors (e.g, Winnipeg) Carpeted vestibule to reduce residue, White cane bars (e.g, Calgary)	No steps (49)
Flooring	Hard materials Non-slippery materials Textured paths and lanes (e.g, Beijing airport)	Hard materials (71) Non-slip material Light-coloured Textured path Braille blocks Use of LED paths, colour coding, tactile information and luminescent materials (57)
Ramps		Whenever possible to limit required efforts for PwD and assistants (70) Limited steepness (49)
Handrails	In main circulation areas, to support mobility and orientation (e.g, Beijing)	
Seating	Reserved seating, placed throughout terminals for respite and waiting areas (e.g, Beijing Airport) Raised for people who have trouble getting up (e.g, Paris CDG)	Regular intervals to reduce walking distance (71)
Meeting points and landmarks	Located near recognizable features, highly signposted, often used for kiosks and assistance counters (e.g, Madrid)	Should be placed to minimize walking distances (71)
Elevators	Braille buttons Voice assistance (e.g, Canton) Mirrors Tactile signs for directional information Strategically placed near staircases and escalators (e.g, Calgary)	Should be placed to minimize walking distances (71)
Escalators and stairs	Placed in high traffic areas (e.g, Denver)	Should be large: Limited space of escalators can cause issues for large mobility aids and during emergencies (39) Tactile signs at both ends should be present to indicate the direction of movement for people with visual impairments (61). Should be placed to minimize walking distances (71)
Vehicles	Wheelchairs rent or free self-service (e.g, Singapore) Electric vehicles Buggies Carts (e.g, Dubai)	
Restroom	Dedicated cubicles in toilets (one or two per general public room) (e.g, Québec) Gender-neutral toilets for personal attendants Increased space for wheelchair circulation Handrails Lowered sink Objects table Coat Hook Lowered hand dryers Lowered water fountain (e.g, Beijing Airport)	Large spaces for wheelchair circulation. Grab bars. Clean spaces for medical procedures (49)

In all the airports surveyed, regulatory exemptions are in place to allow the free circulation of people with assistance dogs. However, it is the passenger's responsibility to inform the airlines and provide the necessary documentation (animal passport and identification documents) to justify the need for a service dog. Many airports, such as Calgary and Dallas's airports, offers pet-friendly outdoor areas, before and after security. However, in numerous cases, those relief areas are too scarce (49).

Despite those features, several challenges are faced by PwD when they navigate airport environment. Firstly, wheelchair users have to check in their wheelchair for transportation (72). For this reason, many airports offer self-service wheelchairs for both indoor and outdoor use, they may not be comfortable and affect individual autonomy (66). In addition, wheelchairs may be damaged during transit. Beside the inconvenience of not having a wheelchair due to damage, insurance procedures in those cases are complicated (49). Also, it is not uncommon to have to change wheelchairs twice: from their personal wheelchair to a wheelchair for use in the airport, and then to a smaller wheelchair for boarding the plane.

Furthermore, passengers have to change vehicles between the different stages of the journey. Besides, access to vehicles varies greatly between airports. However, the vast majority have at least electric vehicles in the terminal waiting areas after security screening.

#### 3.3.5 Information and wayfinding at the airport

Several practices and technologies are in place in airports to allow PwD to access to timely information. Hearing loops, such as those used in Tokyo Haneda Airport (73), enable people with hearing disabilities to obtain crucial information about flights and airport situation. Hearing Loops are assistive listening systems, designed for people wearing hearing aids (T-coil, cochlear implants and streamers), that improve the clarity of information and communication. Also, a number of teletypewriter telephones (typed messages telephone) and video relay services are in place in many airports to facilitate communication between passengers and stakeholders.

Also, airports usually rely on the use of flight information panels, coupled with auditory information on changing situations (cancellation, etc.). Information panels should present data multimodally, without separating situational and contextual information (74). Park et al. (38) point out that most airports aspire to transmit all information to PwD in a lossless, time-efficient and intuitive way (38). However, it can be difficult for PwD to obtain real-time flight information. For example, if important changes occur and are announced audibly, a hearing-impaired person may not have the initiative to reconsult the information board (61, 74).

Interestingly, for some PwD, other passengers can act as a supportive information resource. While these interactions are positive most of the time, discriminatory or marginalizing attitudes or remarks may be made (49, 61).

In addition, Toronto Pearson Airport recommends that people with visual impairments use the Aira application, which enables them to communicate with an assistant via videoconferencing, in real time (75). Another application, based on the use of IBeacon in airports, offers real-time information at every step, to enable route decisions to be made, based on time and distance. The information presented is multimodal (haptic feedback, audio, text) and flexible (e.g, customizable level of detail) (74). However, for indoor navigation systems, vast airport spaces can make it difficult to install beacons covering all possible paths. Indoor systems are also less accurate than outdoor systems, which can be a problem when lanes are narrow or when hazardous installations are nearby (61).

#### 3.3.6 Check in

Several airports, such as Singapore Changi or London Heathrow offers dedicated accessible counters in check-in areas (51, 53). In some airports (e.g, Amsterdam Schiphol, Dubai International) and airlines (e.g, Delta Airline or Qatar Airways) offer priority check-in for people with disability (64, 69). This service allows PwD to avoid long waiting times and complete the check-in with assistance. At all airports, airline or airport employees are present at check-in counters and in queues to offer assistance. Airports increasingly use automatic accessible kiosks and touchscreen, to improve check in efficiency. At Hong Kong international, those devices are adjustable in height and can display larger fonts and contrasts for PwD (76).

Check-in, however, can be challenging for PwD. At every key travel stages, airports are particularly dense in terms of people and traffic. The density of people can hinder access to counters and reserved spaces for PwD (57).

#### 3.3.7 Security screening

The high passenger traffic, the impossibility of using regular scanners, the additional equipment to be checked and the sometimes confined spaces are additional stressors for PwD during security screening (49, 77, 78). To bridge this gap, priority screening and dedicated lanes can be in place, such as Hartsfield-Jackson Atlanta International Airport (79), to avoid traffic and high-waiting times. Furthermore, as in Frankfurt Airport, Germany, PwD can register 72 h prior to the journey to book for a specific time to pass security screening. Also, passengers for whom the usual security procedure may not be possible due to their disability can contact security services for information or to request modified procedures compatible with their conditions, medical devices, service animal, technical aids, etc (72). Furthermore, while security agents usually receive training to assist people with disabilities (78), they cannot provide wheelchair mobility assistance through the security process (72). However, in most airports, PwD can obtain human assistance either from airlines or personal attendants to help with security steps. In Denver Airport, for instance, a pass for non-travelling companions enables PwD to obtain assistance with security and onward areas (54).

Usually, airports’ internal regulations anticipate the additional equipment that PwD will have to carry during screening. For instance, The Canadian Air Transport Security Authority offers extensive information of regulations, exemptions and facilitators for the transportation of medical and other essential items for PwD (80). In Singapore, technical aids are checked separately and an alternative line to metal detectors, with a manual check, is provided for wheelchair users (51).

PwD still face several challenges when going through security screening process. Indeed, they often have to carry out additional documents, as in Kuala Lumpur airport in Malaysia, where passengers are required to keep their medical and identity documents with them at all times (81). Also, the process is also generally more anxiety-provoking and time-consuming, due to the need for a manual search of aids and personal belongings (78). The strain of having to part with one's technical aids and medical devices can be a stress vector for passengers, who fear that their belongings may be lost or damaged in the process (61).

#### 3.3.8 Use facilities and wait at gates

Few airports provide detailed information on assistance to PwD when they are waiting in the gate area. Yet, the ability to use restaurants, bars and other facilities in this area is a key factor in airport accessibility (71). One of the problems at this stage is the transfer and change of responsibility of organizations in human assistance. This pose obvious issues, when people have to use facilities for essential needs, such as having to use the toilet (61). In some Airports, such as Dubai International, a number of accessible facilities are available after security screening, as long as the individual is able to move around the area. Sometimes, other activities in the airport can be supported by assistance services (69). At Heathrow, for example, an attendant can be deployed to help people shopping in the facilities (53).

#### 3.3.9 Boarding and disembarking

In every airport surveyed, airlines are responsible for providing assistance with boarding and disembarking. Individuals must inform companies prior to the journey or during check-in, for any specific required accommodations. In the majority of cases, people board and disembark the aircraft at a dedicated time, before or after other passengers, to ensure they have more time and assistance to reach their seat or get out of the plane, as explained at Paris Charles de Gaulle Airport (82).

Spaces in airplanes almost never accommodate wheelchairs (Holloway et al., 2015). Small type of aisle chairs are used to enable wheelchair users to reach their seat, exit or restrooms (70, 83). However, several people with obesity report negative experience with the inadequacy of airports and airplanes’ chairs (37).

Boarding and disembarking may have to take into account the different possible ways planes dock airports. Most boarding relies on the use of jet bridges, which are movable closed bridges connecting terminals and airports. However, they are not used in every situation, for example, when boarding and disembarking take place directly onto the airfield ground (tarmac). Information on procedures in that case is not available on airport websites. Some airports, such as Toronto Pearson or Kuala Lumpur International, recommend that passengers directly contact airlines for assistance and information with specific boarding and disembarking procedures (10, 84).

#### 3.3.10 Customs and immigration

Few airport websites offer information on accessibility and inclusion at customs and immigration. At Dubai International, *a priori*ty lane with a dedicated counter and a person trained to assist PwD facilitates the declaration process. The scarcity of information on airport websites is not surprising, since those services are managed by immigration and customs agencies. For instance, the Canadian Border Services Agency offers information on priority and alternative lines: they can provide assistance in completing the declaration and documents, accommodate sign language and handle personal items for more extensive clearance (85)

To support independence, it is recommended that PwD be offered the opportunity to use accessible automatic information and service terminals (e.g, customs service terminal) (57, 86). The IATA offers a practical guide to support the implementation and use of automatic border control systems (86). However, those types of technologies can be hard to implement in airports, if they are perceived to be too costly to implement, or if too few people are likely to use them (86).

#### 3.3.11 Managing luggage and personal belongings

Managing luggage during the journey can be a difficult task for people with disability, whom mobility is reduced (37, 49). In addition, they often have to travel with additional equipment, required either for their health or their activities (apnea monitors, personal supplemental oxygen, mobility aids, etc.), that can affect travel in many ways. First, such items take up space that might already be limited (35). Also, people travelling with wheelchairs or other bulky equipment need to plan for their handling. Inconsistencies and not knowing what happens with equipment handled by airport's employees can be stressful for PwD (66).

Luggage assistance is included with human assistance services at most airports. For instance, at Calgary Airport, help is offered as soon as the person arrives at the airport to check in, and when collecting from conveyors after their flight (36).

However, those services need to be planned prior to travel, and might require waiting time at arrival, depending on the destination. Also, this step can be challenging for PwD, when they are alone or when services are unavailable. Finding and lifting bags from conveyors may not always be possible. In some cases, PwD may try to avoid having a checked baggage, to avoid this step altogether (61).

#### 3.3.12 Sustaining travel experience

PwD often express the need to be able to move around airports independently, without depending on constant assistance (61, 86). They need to have access to the same opportunities and channels as other passengers. For them, airports should pay particular attention to the risk of perpetuating the normative transport culture, which allows passengers without disabilities more efficient mobility from the outset (48, 86). However, the way services currently operate can create a sense of marginalization—even humiliation—among PwD (49). For instance, people with visual impairments may feel that their autonomy is being taken away, when assistants present them with a wheelchair (61). Another example is the assumption that people in wheelchairs are unintelligent and dependent (49). Passengers wish that employees would be trained, and use communication strategies that promote their autonomy (67). Also, the use of waiting areas isolated from other passengers can create a sense of exclusion and prevent PwD from exploring and using the various infrastructure offered by airports (86). The way PwD are perceived and conceptualized in airports has also a direct impact on the way they are treated (37, 61, 67). PwD are referred to by many names: special passengers, people with reduced mobility, the disabled, handicapped, challenged passengers, passengers with special needs, People of Determination, people with impairments, old, weak, sick. Budd and Ison (87) recommend standardizing the definition of PwD or persons with reduced mobility, to increase the quality and consistency of services.

Regulations, as well, play a decisive role in improving travel accessibility and experience (61). International organizations such as the International Civil Aviation Organization (ICAO) and the International Air Transport Association (IATA) have taken a stand on the need to accommodate PwD. They emphasize equity in service provision and accessible approaches, particularly at borders (86). In the United States, the Air Carrier Access Act (ACAA) and the Americans with Disabilities Act (ADA) protects people with disabilities from discrimination at all stages of travel (67, 74). Similarly, in Canada, regulations on transport accessibility for PwD are at the heart of strategic planning for international airports, as in Toronto and Calgary (88, 89). In Europe, regulations for the equity and quality of services for PwD, such as the Convention for the Rights of PwD, guarantee free assistance, at all stages of travel (61, 70). Nonetheless, respect and adherence to laws and regulations can be a challenge for PwD. In some cases, laws exist but are little known. For instance, the Department of Transportation regulation guarantee that PwD are subject to the same travel fees, regardless of how they communicate with services. However, Lazar et al. (48) have shown that in many cases, airline agents on the phone do not comply with this obligation, even when it is explained to them. To prevent inconsistencies in laws application and inequities, Budd and Ison (87) recommend that airport companies inform passengers on laws and their responsibilities for travel. They should also participate in the review of laws, and their critics, to prevent abuse of service, and clarify ambiguous regulations.

Every airport puts forward a customer service department, to sustain travel experience. For example, Toronto airport has set up a 24-hour helpline, live chat and contacts phone number. At Tokyo Haneda airport, dedicated customer service agents are deployed at accessible counters (73). Nevertheless, the contact process can sometimes be difficult. The lack of accessibility of some websites can deter PwD from getting in touch with customer service to raise issues (48).

## 4 Discussion

This scoping review highlights many practices and challenges for accessibility and inclusion in airports. At first glance, the challenges and negative experiences of passengers with disabilities may appear paradoxical, considering the large number of measures in place to support their travel experience. However, current practices may underestimate the complexity of air travel (90). Conceptions of accessibility and inclusion can be influenced by visions that may be mistakenly believed to be universal (e.g, Western view of disability). Indeed, air travel has many meanings and purposes and can be pursued in many different ways. This reality is particularly important in airports, which welcome people from all over the world, with very different cultural backgrounds. Some salient challenges experienced by PwD can be deduced from this exploration. On the one hand, they carry a heavy burden when travelling, and experience tensions with airports’ views of their identity. On the other hand, the scale and complexity of airports are a major challenge for stakeholders in supporting PwD.

### 4.1 The burden of travel for PwD

The results show that, while many services exist, passengers who need assistance have to plan their journey to a much greater extent. Indeed, services are not always immediately available to PwD, without reservation. If they are, they often require additional time for deployment. Implicitly, this approach to services, places a great deal of responsibility on PwD in their ability to travel. This vision of accessibility is rooted in current practices. Indeed, travel guides designed for PwD place considerable emphasis on passengers’ and families’ responsibility for travel. Several additional steps are required, and it's up to PwD to prevent setbacks at each step. Many organizations are explicit on this topic: they specify that the individual is responsible, and sometimes decline responsibility for the failure of the trip, if arrangements have not been made by the passenger beforehand. This position is at odds with the recommendations or obligations issued by several governments. Indeed, the legal framework suggests an inclusive approach, where PwD have the same opportunities as any other passenger. In a context where this additional burden can be detrimental to the possibility of making the journey, it is legitimate to think that PwD may effectively not have the same opportunities as other passengers.

Planning is often at the forefront, in a context where the notion of time is critical to air travel. The success of the journey depends in particular on the person's capacity to get through the various stages in a timely manner. The deployment and use of alternative services or channels can lead to additional delays in the journey. Airports are well aware of this, as many insist on the importance of arriving at the airport well in advance. Long waits for an assistant is one example that raises concerns about the management of travel time. The chain of steps involved in air travel is long and complex. The accumulation of additional delays at each stage is a major concern for passengers with disabilities.

These issues are exacerbated by the ambiguity surrounding information on accessibility. The use of generic rather than descriptive adjectives and terms, such as “accessible” without further detail, is common practice on websites. Since people's needs are as unique as they are diverse, each individual is in the best position to assess their own needs. This can hinder PwD's preparation, as they cannot always conclude whether the services in place are sufficient to meet their specific needs. Similarly, few really prepare PwD for the challenges they will face at airports. Most airport organizations present the services they offer, to demonstrate that the journey will be accessible, without acknowledging limitations. Heathrow Airport in London, UK stands out in this respect. The organization conveys information about accessibility, acknowledging the challenges that will accompany the journey (e.g, additional delays, regulatory limitations, etc.) (53).

For the same reason, in addition to increased preparation, passengers with disabilities need to have a higher level of knowledge of air travel (5, 91). The inability of people to anticipate problematic situations that may arise at various stages jeopardizes their ability to travel.

### 4.2 The tension of the PwD vision

One of the main reasons why PwD don't travel is the dehumanizing nature of travel, as described by Darcy (5). The current state of services and their description corroborate this experience. The medical vision, focused on the disability rather than the person, is predominant, both in airports and among external actors involved in services. PwD are categorized in two ways, either by their disability, or by the needs deduced from their disabilities. This disability-centric perspective influences services in several ways.

Firstly, the complexity and uniqueness of PwD are often overlooked. By reducing people to their disability or perceived needs, services are neither personalized nor respectful of passengers’ agency. Rarely can people decide or express what they want in terms of the level of assistance, the route he wishes to take or the places he wishes to visit. Moreover, although passengers’ vision of autonomy varies widely, it is often conceived in just one way, at airports. For example, employees’ level of knowledge about disability can lead to the development of a perception that PwD need to be assisted in everything they do (67). On the other hand, services and infrastructures have a well-defined objective: to complete travel stages. To do so, it is assumed that people need to meet their primary needs, and to mobilize. If these needs are important to the journey, it's possible that other needs are just as important. It is rarely possible for passengers with disabilities to take full advantage of the wide range of activities that airports have to offer. In particular, human assistance services are often well-defined to a specific route, which only allows the completion of travel stages. Also, the approaches are often the same, regardless of the passenger. The unique meaning of travel for the individual is therefore rarely called upon.

Secondly, services are focused on inability, rather than strengths. In many cases, the passenger must inform the organization in advance about the things he or she cannot do (5, 6). Implicitly, many ask PwD to carry out a prior analysis of their functioning, for the different stages of the journey. In addition to the in-depth knowledge of the journey that this requires of the passenger, this upstream perspective encourages the use of compensatory means, which can exacerbate passengers’ feelings of marginalization. Indeed, such approaches are based on the presumption that the individual is at the root of the problem, rather than on the recognition of a possible mismatch between the individual's context and the occupation. As a result, PwD may feel a heightened sense of guilt in the face of travel difficulties.

Also, the vision and practices of accessibility in the airport context can be stigmatizing for PwD. On the one hand, the terms used to designate PwD often have an unfavourable connotation in relation to the person's abilities. The use of terms such as “weak, challenged, sick” is indicative of the objectives pursued by services and infrastructures. Consequently, employees interact with these people from that perspective, creating, unwillingly, a dehumanizing and stigmatizing experience (61). On the other hand, categorizing people's needs leads to the use of disability-related labels. Many PwD do not identify themselves as “people with reduced mobility,” even though this is the term most often used at airports around the world. In addition to not accurately representing PwD, this term can lead people to believe that PwD only have mobility needs. In fact, a very significant proportion of airport services are designed to support PwD mobility. Services developed to meet other needs are rarer.

Cole et al. (49) warn the industry that meeting the minimum requirement of the law is not enough. Better awareness and education on PwD are required to tackle the stigma and devise more inclusive approaches. Critical thinking about PwD is required to avoid insensitive or discriminatory practices (49).

Increasingly, airport organizations are expressing a desire to change and redefine this vision. Toronto Airport, for example, explicitly makes this distinction in its position statement on accessibility: “We recognize that passengers and employees don't need to adapt to have their needs met; we do” (92). Passengers living with disabilities insist on the importance of bringing this critical reflection to the development and planning of accessibility practices (5).

### 4.3 Organizational challenges at airports

The magnitude and complexity of airports are a major challenge for the organization of services and infrastructure. Many stakeholders are required to ensure the international reach of airports. Numerous airlines, airport operators and third-party companies have to work together to make travel possible. Each of these organizations has to deal with different realities, objectives and sometimes regulatory frameworks. This reality of shared responsibility can hinder service synergy. The many different organizations working on accessibility have mandates defined for specific actions or areas (93). As mentioned before, for example, airport organizations are responsible for getting people to the gates, while airlines are responsible for boarding and disembarking. Elsewhere, as in Calgary, some airlines take charge of PwD as soon as they check in. The need to deal with heterogeneous operating modes can hamper continuity of service (93). Also, there is little evidence of sustained communication between these stakeholders: usually, it's the passenger who has to do the bridging (61). This complexity is exacerbated by the many stages of travel, for which PwD need support. Because of the way stakeholders interact, the PwD travel process is fragile. It only takes a perturbation at one stage of the journey to jeopardize the entire planning and organization of the other stages. Safeguards are not always in place to compensate for the uncertainties of the journey, and service providers do not always assume responsibility for making the journey possible at all costs. Moreover, this state of communication is conducive to service errors. Certain needs may be misinterpreted or poorly conveyed, resulting in a reduction in service quality (5, 61). Also, transitions between different services are often criticized in the passenger experience. The lack of a bridge between the transfer of responsibility is at the root of major discomfort situations for passengers. For example, they may have to wait for long periods, with no assistance available for their basic needs. In addition, airports are home to a complex network of human resources. Assisting PwD requires specific knowledge and skills. Maintaining these skills is a challenge at many airports, which have to cope with high staff turnover, limited resources (49) and poor access to skilled labor. These training and skills issues can also diminish the benefits of initiatives designed to promote inclusion. For example, programs aimed at increasing the visibility of people with invisible disabilities can be compromised if assistants have little knowledge of the approaches to be favoured.

More and more airport organizations are talking about inclusion. In many countries, legislation is pushing organizations to think critically about equal opportunities for PwD. Airports often wish not only to meet their legal obligations, but also to develop the quality of their services and travel experience. Major challenges remain in respecting passenger rights (48), and more generally in service quality. However, the various initiatives, calls for projects and partnerships set up by airports demonstrate their interest in narrowing this gap.

### 4.4 Strengths and limitations of the study

This study has several strengths. Firstly, the methodological choices, informed by the scope review method, contribute to the study's relevance and credibility. The process of identifying and selecting articles was carried out by two people, double-blind. Disagreements were discussed ensuring accuracy and comprehensiveness. Secondly, systematic consultation of the websites of airport organizations greatly contributed to data richness and comprehensiveness. Airport organizations are the main resources for obtaining information on accessibility services and infrastructures. Their inclusion was essential to obtain an accurate picture of the state of accessibility and inclusion in international airports. Moreover, the varied origins of the airports also enabled us to highlight critical differences between the various international airports. At the same time, we took a broad look at the contextual aspects that play a role in accessibility. By considering all stages of the journey, it was possible to highlight important gaps, transversal to accessibility and inclusion practices (e.g, issues of disability vision and organizational challenges).

Several limitations should also be noted. In particular, we did not systematically include Google search results when identifying documents. In the search for accessibility practices, the vast majority of results from the Google search engine refer to international airport websites. Since the Google search is conducted from Canada, the search favoured results related to North American airports. However, for the purposes of this study, we wanted to consider airports in terms of their activities, rather than their location. With this in mind, we chose to proceed systematically by directly identifying the sites of the world's busiest international airports. Also, as mentioned previously, accessibility information on websites is sometimes ambiguous and vague. Generic terms such as “accessible” are used to include a range of features and accommodations that are not described on the sites. For this reason, it was not possible to establish clear data on the frequency of arrangements and services as part of this scoping review. Such results could have raised issues of data accuracy, for airports that do not specify or whose information is too general to conclude on the presence or absence of an accessibility feature.

Furthermore, only scientific literature in English and French was considered. It is likely that accessibility or inclusion practices are presented in documents in other languages. Secondly, few data focus on the experience of passengers with disabilities. Although the aim of this study was to highlight accessibility practices, understanding the experience of passengers with disabilities is equally important in improving services. Similarly, understanding the needs and experience of individuals is crucial to taking a stance on the occupational participation of passengers with disabilities. To this end, Prajapati et al. (94) are currently conducting a scoping review on the experience of PwD when flying.

## 5 Conclusion

Air travel is very important for the well-being of everyone, and, of course, of PwD, but also for their fundamental right to equal opportunities. Air travel is increasingly becoming a common means of transport, sometimes essential to many important roles in people's lives. Even today, a considerable number of PwD do not travel, due to the difficulties anticipated in the process (91). For this reason, various organizations are working on several fronts, to support access to travel for these individuals. This study has painted a picture of accessibility and inclusion in airports around the world. A wide variety of practices, at many contextual levels, were identified. The results are an important step towards understanding the main challenges affecting the occupational participation of PwD when travelling through international airports. Several possible directions for future research were raised. In particular, gaps relating to the burden of travel for PwDs, their vision by the various stakeholders and organizational management were highlighted. Narrowing these gaps is crucial, given that it's not enough for airports to be “accessible”, but that they must offer PwD a satisfying and humane travel experience.

For this reason, this study is the first step in a larger project, funded by Canadian Accessibility Standards, aimed at improving inclusive accessibility in Canadian airports. We will be accompanying PwD of various profiles through the various stages of their journey, to gather their perceptions of the travel experience. The results will be used in conjunction with this study to formulate recommendations and solutions for inclusive accessibility.

## Data Availability

The raw data supporting the conclusions of this article will be made available by the authors, without undue reservation.

## Appendix A: Scientific literature research terminology

### All free terms were searched within titles and abstracts

#### Medline (via ebsco)

(disab* OR “special need*” OR incapacit* OR “physical disorder*” OR “reduced mobility” OR “physical* challenge*” OR (MH “Disabled Persons+”) OR visual* impair* OR vision OR blind* OR “deaf*” OR hearing* OR (MH “Sensation Disorders+”) OR intellectual OR “mental* deficienc*” OR “Developmental” OR “mental* ill*” OR “mental* disorder**” OR “mental* health disorder*” OR “psychiatric disorder*” OR “psychiatr* ill*” OR neurodivergen* OR neuro-divergen* OR autism OR autistic OR cognitive OR dementia OR (MH “Mental Disorders+”) OR elder* OR older OR senior OR aged OR (MH “Aged+”) OR (MH “Aging+”) OR Obes* OR (MH “Obesity+”)) **AND** (OR modif* OR “access* program*” OR “access* measure*” OR “universal access*” OR design* OR “assistive technolog*” OR “architectur* access*” OR adapt* OR Signage OR (MH “Architecture+”) OR (MH “Self-Help Devices+”) OR Inclusiv* OR discriminat* OR Respect* OR Equity OR Justice OR tolerance OR prejudice OR (MH “Social Inclusion”) OR (MH “Social Discrimination”) OR (MH “Respect”) OR (MH “Prejudice”) OR (MH “Social Justice”) OR (MH “Social Isolation”) OR Guideline* OR “Framework” OR “Frame of reference” OR “Best practice*” OR recommendation* OR polic* OR Rule* OR Regulation* OR (MH “Models, Theoretical”) OR (MH “Guidelines as Topic+”) OR (MH “Policy”) OR (MH “Health Planning+”) OR ([Neutral* OR equity OR (MH “Gender Equity”)] AND [bathroom* OR restroom* OR Utilit* OR Facilit* OR (MH “Toilet Facilities”)])) **AND (**Accessib* OR Orientation OR Mobil* OR (MH “Architectural Accessibility”) OR (MH “Spatial Navigation”) OR inclusiv* OR participation OR (MH “Social Participation”) OR (MH “Community Participation”) OR use OR usability OR utilization OR capab* OR capacity OR ability OR adequacy OR appropriateness OR experience) **AND (**airport* OR “air transport*” OR “airline*” OR aircraft OR (MH “Airports”) OR (MH “Aviation+”) OR (MH “Air Travel”)

#### CINAHL (via ebsco)

(disab* OR “special need*” OR incapacit* OR “physical disorder*” OR “reduced mobility” OR “physical* challenge*” OR (MH “Persons with Disabilities+”) OR visual* impair* OR vision OR blind* OR “deaf*” OR hearing* OR (MH “Sensation Disorders+”) OR intellectual OR “mental* deficienc*” OR “Developmental” OR “mental* ill*” OR “mental* disorder**” OR “mental* health disorder*” OR “psychiatric disorder*” OR “psychiatr* ill*” OR neurodivergen* OR neuro-divergen* OR autism OR autistic OR cognitive OR dementia OR (MH “Behavioral and Mental Disorders+”) OR elder* OR older OR senior OR aged OR (MH “Aged+”) OR (MH “Aging+”) OR Obes* OR (MH “Obesity+”)) **AND** ((modif* OR “access* program*” OR “access* measure*” OR “universal access*” OR design* OR “assistive technolog*” OR “architectur* access*” OR adapt* OR Signage OR (MH “Facility Design and Construction”) OR (MH “Assistive Technology Devices”) OR (MH “Signage”) OR Inclusiv* OR discriminat* OR Respect* OR Equity OR Justice OR tolerance OR prejudice OR (MH “Social Inclusion”) OR (MH “Attitude to Disability”) OR (MH “Respect”) OR (MH “Prejudice”) OR (MH “Social Justice”) OR (MH “Occupational Justice”) OR Guideline* OR Framework” OR “Frame or reference” OR “Best practice*” OR recommendation* OR polic* OR Rule* OR Regulation* OR (MH “Practice Guidelines”) OR (MH “Conceptual Framework”) OR (MH “Rules and Regulations”) OR (MH “Government Regulations”) OR (MH “Public Policy”) OR ([Neutral* OR equity OR (MH “Gender Equality”)] AND [bathroom* OR restroom* OR Utilit* OR Facilit* OR (MH “Toilet Facilities”)]) **AND** [Accessibility OR Orientation OR Mobil* OR inclusiv* OR participation OR (MH “Social Participation”) OR use OR usability OR utilization OR capab* OR capacity OR ability OR adequacy OR appropriateness OR experience] **AND** (airport* OR “air transport*” OR “airline*” OR aircraft OR (MH “Aviation”) OR (MH “Aircraft”) OR (MH “Travel+”))

#### Avery index to architectural periodicals

(disab* OR “special need*” OR incapacit* OR “physical disorder*” OR “reduced mobility” OR “physical* challenge*” OR (DE “People with disabilities”) OR (DE “Public health”) OR (DE"Architecture and the physically handicapped”) OR visual* impair* OR vision OR blind* OR “deaf*” OR hearing* OR (DE “Hearing impaired”) OR (DE “Visually impaired”) OR intellectual OR “mental* deficienc*” OR “Developmental” OR “mental* ill*” OR “mental* disorder**” OR “mental* health disorder*” OR “psychiatric disorder*” OR “psychiatr* ill*” OR neurodivergen* OR neuro-divergen* OR autism OR autistic OR cognitive OR dementia OR (DE “Autism”) OR elder* OR older OR senior OR aged OR (DE “Aged”) OR Obes* OR (DE “Obesity”)) AND (modif* OR “access* program*” OR “access* measure*” OR “universal access*” OR design* OR “assistive technolog*” OR “architectur* access*” OR adapt* OR Signage OR (DE “Universal design”) OR (DE “Barrier-free design”) OR Inclusiv* OR discriminat* OR Respect* OR Equity OR Justice OR tolerance OR prejudice OR (DE “Social justice”) OR Guideline* OR Framework” OR “Frame of reference” OR “Best practice*” OR recommendation* OR polic* OR Rule* OR Regulation* OR (DE “Government policy”) OR ((Neutral* OR equity) AND [bathroom* OR restroom* OR Utilit* OR Facilit* OR (DE “Rest rooms”)]) AND (Accessib* OR Orientation OR Mobil* OR (DE “Access) OR (DE “Access to airports”) OR inclusiv* OR participation OR (DE “Citizen participation”) OR (DE “User-centered system design”) OR use OR usability OR utilization OR capab* OR capacity OR ability OR adequacy OR appropriateness OR experience) AND (airport* OR “air transport*” OR “airline*” OR aircraft OR (DE “Choice of transportation”) OR (DE “Airports—Buildings”))

#### Web of science

(disab* OR “special need*” OR incapacit* OR “physical disorder*” OR “reduced mobility” OR “physical* challenge*” OR visual* impair* OR vision OR blind* OR “deaf*” OR hearing* OR intellectual OR “mental* deficienc*” OR “Developmental” OR “mental* ill*” OR “mental* disorder**” OR “mental* health disorder*” OR “psychiatric disorder*” OR “psychiatr* ill*” OR neurodivergen* OR neuro-divergen* OR autism OR autistic OR cognitive OR dementia OR elder* OR older OR senior OR aged OR Obes*) AND (modif* OR “access* program*” OR “access* measure*” OR “universal access*” OR design* OR “assistive technolog*” OR “architectur* access*” OR adapt* OR Signage OR Inclusiv* OR discriminat* OR Respect* OR Equity OR Justice OR tolerance OR prejudice OR Guideline* OR Framework” OR “Frame or reference” OR “Best practice*” OR recommendation* OR polic* OR Rule* OR Regulation*) OR ((Neutral* OR Equity) AND (bathroom* OR restroom* OR Utilit* OR Facilit*))) AND (Accessibility OR Orientation OR Mobil* OR inclusiv* OR participation OR use OR usability OR utilization OR capab* OR capacity OR ability OR adequacy OR appropriateness OR experience) AND (airport* OR “air transport*” OR “airline*” OR aircraft)

## Appendix B: Airports’ websites free search terms

**Table:**

Disability/ies	Special	Impairment/impaired	Needs
Older/old/aged	obesity	Access	Accessibility
Assistance	Assistive	Accommodation/accommodate	Equity
Inclusive/vity	Discrimination	Help	Customer service
Service.S	Mobility	Wayfinding	Complaint

## Appendix C: data extraction grid sample (excel)

The following table contains some non-exhaustive examples of data extracted for a scientific article, for illustrative purposes.

**Table:**

Reviewer name	DG
Title	Airports and ageing passengers: A study of the UK
Authors	Anne Graham, Lucy Budd, Stephen Ison, Andrew Timmis
Year	2019
Study location	UK
Study objective	(…) the aim of this paper is to undertake an exploratory analysis of ageing passengers at UK airports.
Study design	Case study
Population	Older adults
Needs addressed (explicitly in the text)	much of the extant literature on transport and ageing focuses on driving cessation, public transport use and the role of active travel in supporting healthy older age (…) while the impacts of ageing on air travel are hitherto comparatively unexplored.
Purpose of the practice(s) described	N/A
Micro (factors and practices)	(…) Age-related hearing and sight loss, as well as mobility and cognitive impairments, can create challenges associated with navigating new environments and negotiating the procedural logic of air travel, while advances in airport automation may generate anxiety and confusion among an older generation who have not grown up with the technologies and who are consequently not familiar with their purpose and operation. (…) allegations of pre-booked assistance failing to meet user needs, inappropriate equipment, inadequate staffing levels and poor customer service standards often reported (Buckley, 2017). […]
Meso (factors and practices)	In the United States, the Transportation Security Administration (TSA) (2019) has introduced special security screening protocols for passengers aged 75 and over which negates the need for older travellers to divest of shoes and clothing during the security search. (…) priority boarding and disembarkation from the aircraft In response, some airports are seeking to become ,”Dementia Friends“ by providing staff with additional training and providing lanyards to allow customers self-identity if they think they would benefit from additional support. […]
Macro (factors and practices)	Within the European Union, all passengers with a disability (whether physical, cognitive or communicative) or reduced mobility (irrespective of age) are legally entitled to support or “Special Assistance” whilst travelling by land, water and air. EC Regulation 1107/2006 states that all EU airports handling over 150,000 passengers a year must provide, free of charge, help and assistance to wheelchair users, older and elderly travellers, and those with communication, social interaction and “hidden” disabilities including autism and dementia. […]
Link to participation (if any)	the latest CAA tracker consumer survey found that 43% of the passengers who requested assistance on their last trip did so for the first time (CAA, 2019b). This survey found that 76%. Those receiving assistance were very satisfied or fairly satisfied with services at the UK airport on departure, and 69% on their arrival back, but satisfaction with the overall flying experience for those with disabilities had decreased from 82% in 2016 to 77% in 2019 (compared to 90% to 81% for the total market) […]
Disability perspective (term used, person or disability centric)	There is a popular misconception that the ageing traveller market consists of frail old people in wheelchairs or with walking sticks. This is incorrect, especially as these travellers make up a number of diverse and heterogeneous consumer groups (Nielsen 2014; Alén et al, 2016).
Research team member reflections and comments	The authors directly support the next steps of our project: “An important area for future work would include in-depth qualitative studies of senior passengers’ experiences of using airports. This would provide both much needed and valuable insight into their needs and a greater understanding of the behaviour of this passenger segment.”
